# microRNA let-7c is essential for the anisomycin-elicited apoptosis in Jurkat T cells by linking JNK1/2 to AP-1/STAT1/STAT3 signaling

**DOI:** 10.1038/srep24434

**Published:** 2016-04-18

**Authors:** Zhiwei Zhou, Xijian Lu, Jin Wang, Jia Xiao, Jing Liu, Feiyue Xing

**Affiliations:** 1 Institute of Tissue Transplantation and Immunology, Department of Immunobiology, Jinan University, Guangzhou 510632, China; 2 Key Laboratory of Functional Protein Research of Guangdong Higher Education Institutes, Jinan University, Guangzhou 510632, China; 3 Department of Stomatology, Jinan University, Guangzhou 510632, China

## Abstract

Anisomycin, an antibiotic produced by *Streptomyces griseolus*, strongly induces apoptosis in various tumor cells *in vitro*, superior dramatically to adriamycin. The present study aims to elucidate its detailed mechanistic process. The results showed that anisomycin sufficiently promoted the apoptosis in human leukemic Jurkat T cells at a quite low dose. microRNA let-7c (let-7c) contributed to the anisomycin-induced apoptosis, which could be abrogated by the inactivation of JNK signaling. The let-7c over-expression and the addition of its mimics facilitated the activation of AP-1, STAT1 and Bim by linking JNK1/2 to AP-1/STAT1, but rather inhibited the activation of STAT3 and Bcl-xL by connecting JNK1/2 to STAT3, followed by the augmented apoptosis in the cells. The let-7c deficiency reduced the AP-1, STAT1 and Bim activities, and enhanced the STAT3 and Bcl-xL, alleviating the anisomycin-induced apoptosis. The knockdown of the *bim* gene repressed the anisomycin-boosted apoptosis through the attenuation of the active Bak and Bax. The findings indicate for the first time that miR let-7c is essential for the anisomycin-triggered apoptosis by linking JNK1/2 to AP-1/STAT1/STAT3/Bim/Bcl-xL/Bax/Bak signaling. This provides a novel insight into the mechanism by which anisomycin leads to the tumor cell apoptosis, potentially laying the foundations for its development and clinical application.

The induction of apoptosis in cancer cells is one of key strategies for cancer therapy^1^,^2^. Apoptosis is a highly regulated form of the cell death characterized by the cell shrinkage, chromatin condensation, membrane blebbing and DNA fragmentation^3^. There are three major pathways for the apoptosis: a death receptor-dependent pathway, a mitochondria-dependent pathway, and an endoplasmic reticulum stress-mediated pathway^4^,^5^. The Bcl-2 family is subdivided into three main categories (based on regions of Bcl-2 homology and function), containing the anti-apoptotic multi-domain (Bcl-2, Mcl-1 and Bcl-xL), the pro-apoptotic multi-domain (Bax and Bak), and the pro-apoptotic BH3-only (Bad, Bid, Bim and PUMA), respectively^6^. A slight change in the dynamic balance of these proteins, regulated at the transcriptional or posttranslational levels, may either inhibit or promote the apoptosis^7^,^8^.

Anisomycin [2-(p-methoxybenzyl)-3,4-pyrrolidinediol-3-acetate] is a pyrrolidine antibiotic purified from the *Streptomyces griseolus* and known to inhibit the protein synthesis by binding to the 60S ribosomal subunits and blocking the peptide bond formation^9^,^10^. It is reported that the anisomycin induces the apoptosis in various human cancer cell lines, such as the promyelocytic leukemia, lymphoma U937, colon adenocarcinoma and the glioblastoma^11^,^12^,^13^,^14^,^15^. We also find that anisomycin strongly promotes the apoptosis in Ehrlich ascites carcinoma cells and colon adenocarcinoma CT26 cells *in vitro* and *in vivo*^16^. The capability of the anisomycin to damage the 28S ribosomal RNA leads to the ribotoxic stress responses that, in turn, stimulate the intra-cellular sentinel signaling pathways, especially the stress-activated mitogen-activated protein kinase (MAPK) subtypes^14^,^17^. Several studies have shown that the anisomycin induces the apoptosis in cancer cells by activating the c-Jun N-terminal kinases (JNKs) or/and p38 MAPK^11^,^12^,^13^,^14^,^15^. The anisomycin exposure always activates the JNK in both normal and malignant cells^11^,^12^,^13^,^14^,^15^,^18^,^19^,^20^,^21^,^22^. However, the downstream targets of JNK in the anisomycin-treated cells remains unclear.

MicroRNAs are small (about 18–24 nucleotides) noncoding RNAs that negatively regulate the gene expression post-transcriptionally by binding to specific mRNA targets and promoting their degradation and/or translational inhibition^23^. Due to their abundant presence, there is mounting evidence suggesting that miRNAs play pivotal roles in a wide spectrum of the biological processes, including the apoptosis^24^. It has been reported that some miRNAs, such as miR-133, miR-155, miR-204, miR-296 and miR-337, are antiapoptotic, while others, including miR-15 and miR-16, are pro-apoptotic^25^,^26^,^27^. Therefore, we wonder whether these miRNAs are correlated to JNK signaling. This study is to elucidate a detailed mechanistic process of the anisomycin-induced cell apoptosis.

## Results

### Low dose of anisomycin induces the apoptosis in Jurkat T cells

**Figure 1:**
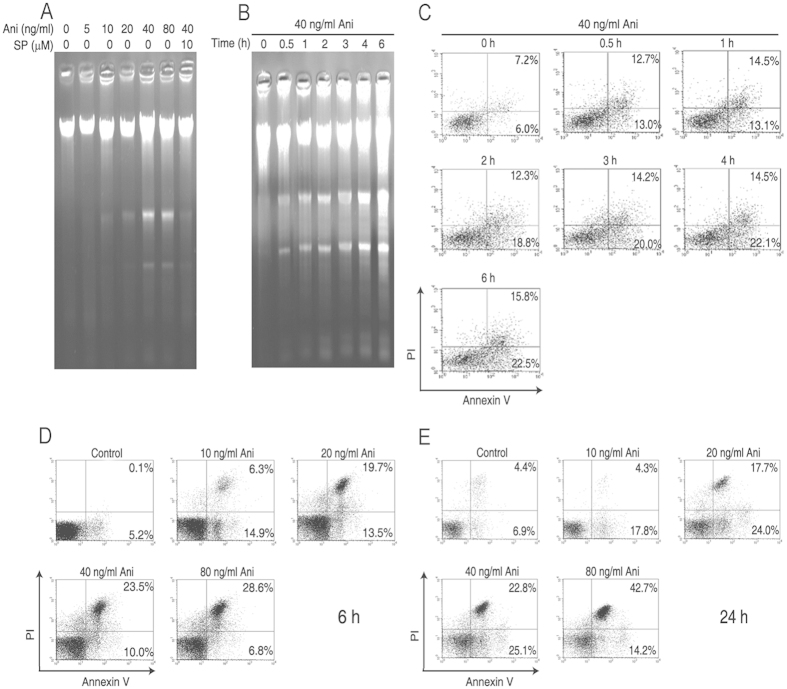
Low dose anisomycin strongly induces the apoptosis in Jurkat T cells. Two hours after pre-incubated with or without 10 μM SP600125 (SP) or 20 μM PD98059 (PD), Jurkat T cells were treated with the indicated concentrations of anisomycin (Ani) for 6 h or 24 h or with 40 ng/ml of anisomycin for the indicated time periods. (**A**,**B**) DNA fragmentation in the treated cells was analyzed by 1% agarose gel electrophoresis. (**C**–**E**) Apoptotic proportion in the treated cells was assayed by flow cytometry using annexin-V/PI double staining. The results show the typical experiment, which has been repeated three times.

As shown in Fig. 1A,B, DNA fragmentations in the cells were gradually increased with the extended exposure time of anisomycin (Ani) or the enhancing concentrations of anisomycin, which was rescued by SP600125 (SP) (Anthra[1,9-cd]pyrazol-6-(2H)-one), an inhibitor for not only JNK, but also MKK4 and MKK7 that are upstream kinases of the JNK. Consistent with the trend of DNA fragmentation alteration, the percentages of the anisomycin-induced apoptotic cells were also increased in a time- or dose-dependent manner (Fig. 1C–E). The percentage of 40 ng/ml anisomycin-induced apoptotic cells reached 47.9% at 24 h after the treatment. These results demonstrate that anisomycin strongly induces the apoptosis in Jurkat T cells through the JNK signaling.

### JNK1/2 signaling plays a major role in the MAPK-mediated apoptosis by anisomycin

**Figure 2:**
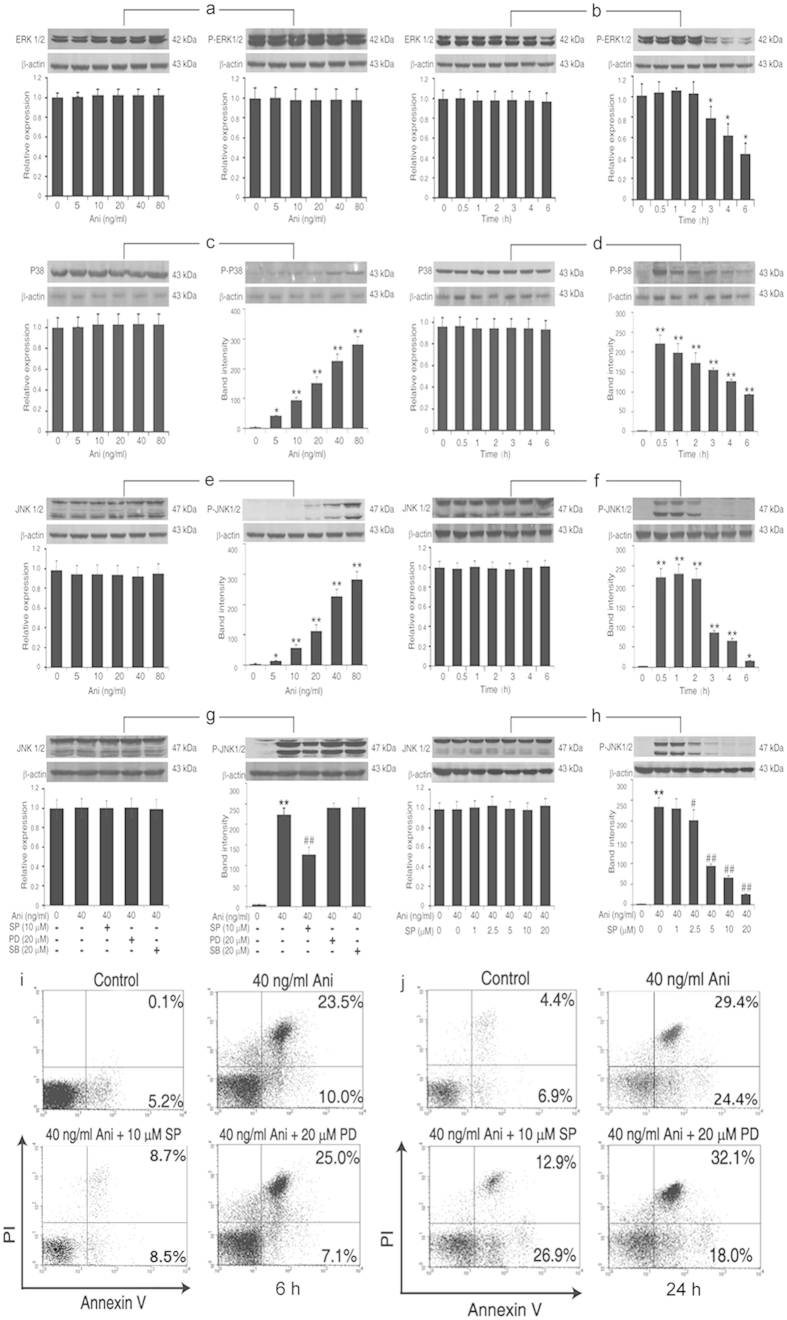
JNK1/2 signaling plays the major role in the MAPK-mediated apoptosis in Jurkat T cells by anisomycin. (**A**–**F**) Jurkat T cells were treated with the increasing concentrations of anisomycin for 1 h or with 40 ng/ml of anisomycin at the indicated time. Following the treatment, the expressions of ERK1/2, P-ERK1/2 (**A**,**B**), p38, P-p38 (**C**,**D**), JNK1/2 and P-JNK1/2 (**E**,**F**) proteins were determined by Western blotting. (**G**,**H**) The cells were pretreated with 10 μM SP600125 (SP), 20 μM PD98059 (PD), 20 μM SB203580 (SB) or the increasing concentrations of SP600125 before the exposure to 40 ng/ml of anisomycin. Then, the expressions of JNK1/2 and P-JNK1/2 were examined by Western blotting. (**I**,**J**) Simultaneously, the apoptotic phenotypes of the cells treated above were observed using annexin-V/PI double staining under the flow cytometer. Data are presented as the mean ± SD of three independent experiments. *P < 0.05, ** *P* < 0.01 *vs*. the untreated control, ^#^*P* < 0.05, ^##^*P* < 0.01 *vs*. the 40 ng/ml anisomycin group.

As shown in Fig. 2, following the incremental anisomycin treatments, the levels of phosphorylated-ERK1/2 (P-ERK1/2) were unchanged, whereas phosphorylated-p38 (P-p38) and -JNK1/2 (P-JNK1/2) were markedly increased with the enhancing doses of anisomycin (0–80 ng/ml). The JNK1/2 activation was more significant than that of P-p38, but no significant changes of non-phosphorylated-ERK1/2, -p38 and -JNK1/2 levels were observed, suggesting that JNK1/2 presents a predominant role in the cell apoptosis induced by anisomycin (Fig. 2A,C,E). Except for P-ERK1/2, P-p38 and P-JNK1/2 were rapidly increased at 0.5 h after the anisomycin exposure. However, the anisomycin treatment did not alter ERK1/2, p38 and JNK 1/2 levels in Jurkat T cells (Fig. 2B,D,F). The levels of the P-JNK1/2 were attenuated only with 10 μM SP600125, but not with PD98059, an inhibitor specific to MEK1/2 that is an upstream kinase of ERK1/2, or SB203580, a p38 MAPK Inhibitor. Moreover, the anisomycin-enhanced P-JNK1/2 expression was obviously down-regulated with the increasing concentrations of SP600125 in a dose-dependent manner (Fig. 2G,H). We also noted that the change of the P-JNK1/2 in the cells was more obvious than that of the P-p38 using an identical dose of anisomycin. Simultaneously, the apoptotic cells induced by anisomycin were rescued by SP600125, rather than by PD98059 (Fig. 2I,J). These results further indicate that the JNK signaling, but not the ERK1/2 signaling, contributes to the anisomycin-induced cell apoptosis. Its role is more significant than that of the p38 signaling.

### Anisomycin facilitates the apoptosis *via* the activation of the JNK/Bim/Bcl-xL pathway

**Figure 3:**
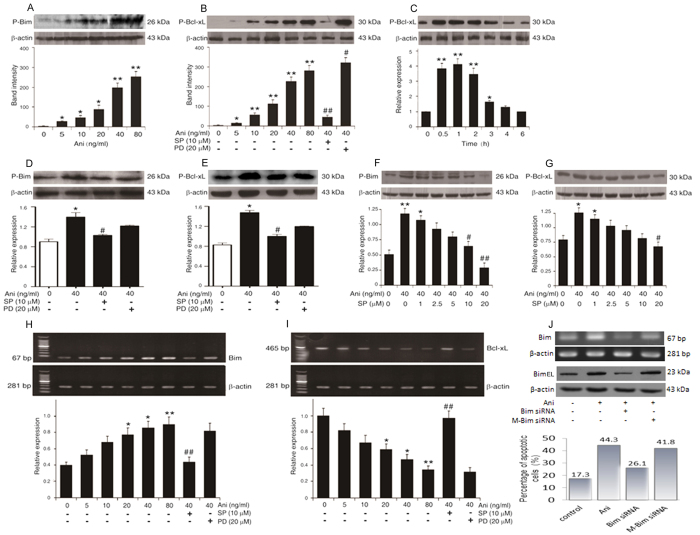
Anisomycin promoted the apoptosis of Jurkat T cells through the JNK-dependent activation of Bim/Bcl-xL. Jurkat T cells were pre-incubated with 10 μM SP600125 (SP), 20 μM PD98059 (PD) or the increasing concentrations of SP600125 for 2 h before treated with the indicated concentrations of anisomycin (Ani) for 6 h. Additionally, the cells were treated with 40 ng/ml of anisomycin at the indicated time points. (**A**–**G**) The expression levels of P-Bim and P-Bcl-xL were assessed by Western blot analysis. (**H**,**I**) The alterations of Bim and Bcl-xL mRNAs were evaluated by RT-PCR. (**J**) Furthermore, Bim-targeting siRNA was used to knock down the *bim* gene to confirm the correlation of its mRNA and protein levels with the cell apoptosis. Data are presented as the mean ± SD of three independent experiments for (**A**–**I**). *P < 0.05, ** *P* < 0.01 *vs*. the untreated control, ^#^*P* < 0.05, ^##^*P* < 0.01 *vs*. the 40 ng/ml of anisomycin alone.

As shown in Fig. 3A–C, the expressions of both P-Bcl-xl and P-Bim proteins were significantly up-regulated with the enhancing concentrations of anisomycin, presenting a dose- or time-related relationship. These changes could be reversed by SP600125, nor PD98059 (Fig. 3B,D–G). Moreover, the expressions of both the P-Bcl-xl and P-Bim proteins induced by anisomycin were obviously down-regulated with the increasing concentrations of SP600125 in a dose-dependent manner (Fig. 3F,G). The Bim mRNA expression was significantly increased with the increasing concentrations of anisomycin in a dose-dependent manner, whereas the Bcl-xL mRNA was obviously decreased with the incremental anisomycin concentrations in a dose-dependent manner (Fig. 3H,I). When the *bim* gene was knocked down with the Bim-targeting siRNA, the process of the anisomycin-induced cell apoptosis might be blocked, following the reduction of Bim mRNA and protein (Fig. 3J). These results strongly indicate that the anisomycin-promoted apoptosis in Jurkat T cells through the JNK-dependent activation of Bim/Bcl-xL.

### AP-1 participates in the JNK/Bim/Bcl-xL signaling-mediated apoptosis by anisomycin

**Figure 4:**
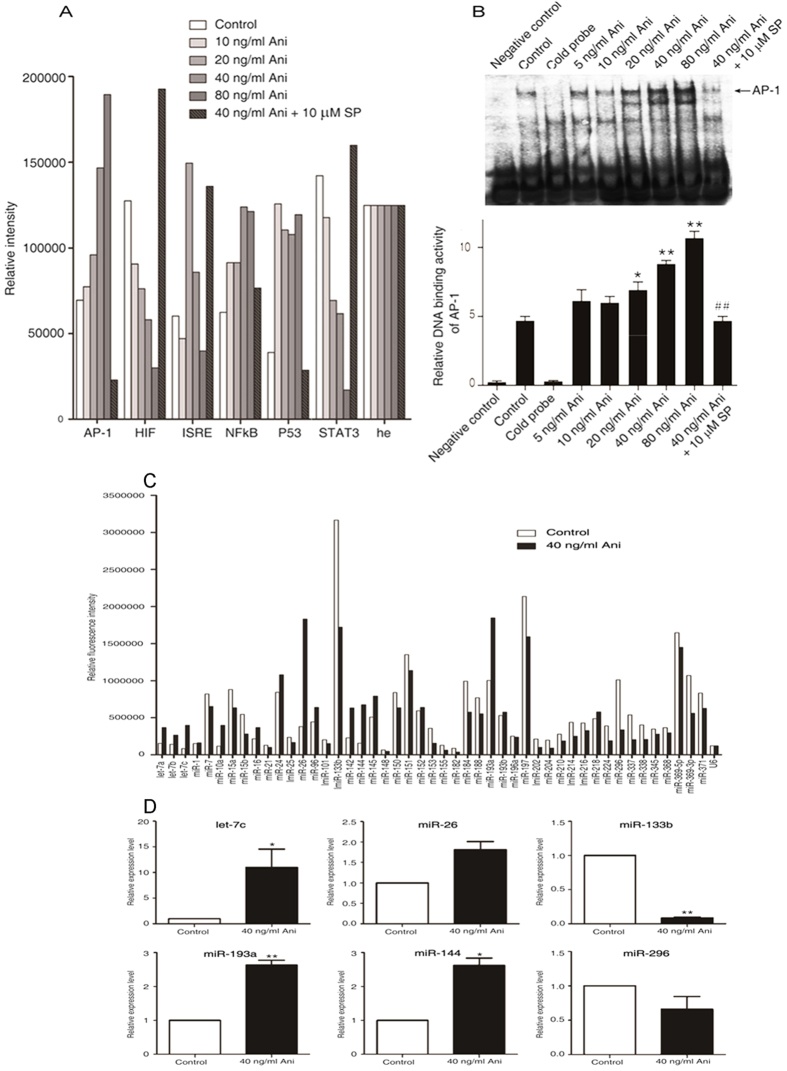
Anisomycin significantly increases the expression of miRNA let-7c in the JNK/AP-1-induced apoptosis of Jurkat T cells. Two hours after pre-incubation with 10 μM SP600125, Jurkat T cells were treated with the increasing concentrations of anisomycin for 6 h (**A**,**B**). (**A**) The activities of the six apoptosis-associated transcriptional factors, AP-1, HIF, ISRE, NF-κB, P53 and STAT3, were measured using the TF Reporter Plate Array. (**B**) AP-1 DNA binding activity was tested by the electrophoretic mobility shift assay. On the other hand, the cells were treated with or without 40 ng/ml of anisomycin for 24 h (**C**,**D**). (**C**) miRNA plate array analysis for 47 apoptosis-associated miRNAs was performed with the RNA extracts from the treated Jurkat T cells. (**D**) The real-time quantitative PCR analysis of differentially expressed six miRNAs, including *miR let-7c*, miR-26, miR-133b, miR-193a, miR-144 and miR-296, was performed to further validate the microarray results. More than triplicate assays were carried out for each RNA sample. For the (**A**,**B**), the data are presented as the mean ± SD of three independent experiments. *p < 0.05 and **p < 0.01 *vs*. the untreated control, ^##^*p* < 0.01 *vs*. the 40 ng/ml of anisomycin group. For the (**C**,**D**), The data are shown as the fold changes of the miRNA levels in the anisomycin-treated group relative to the control group, *p < 0.05, **p < 0.01.

It was also reported that anisomycin strongly induces the transcription of several immediate–early genes as a result of its potent activation of the MAP kinases^18^,^28^,^29^,^30^. As shown in Fig. 4A, the activities of AP-1 (activation protein-1) and NF-κB were significantly up-regulated in a dose-dependent manner, whereas the activities of HIF-1(human hypoxia inducible factor) and STAT3 (signal transducers and activators of transcription 3) were obviously down-regulated with the enhancing concentrations of anisomycin. Moreover, the low dose of anisomycin was sufficient to up-regulate the P53 transcriptional activity. Interestingly, the ISRE (interferon stimulated response element) activity was increased with the lower anisomycin dose, but rather decreased with the higher dose. All the above-mentioned changes could be reversed by the pretreatment with the JNK inhibitor SP600125. In comparison with the control, the AP-1 DNA-binding activity was significantly augmented with the enhancing concentrations of anisomycin. JNK inhibition protected against the anisomycin-induced AP-1 binding activities (Fig. 4B). Taken together, these findings indicate that AP-1 participates in the JNK/Bcl-xL/Bim signaling-mediated apoptosis in Jurkat T cells by anisomycin.

### miRNA let-7c regulates the downstream molecules in the anisomycin-stimulated JNK signaling through AP-1/STAT1/STAT3

Among the individual miRNAs represented on the microarray, six of the apoptosis-associated miRNAs, including let-7a, let-7c, miR-10a, miR-26, miR-142 and miR-144, were significantly up-regulated by anisomycin. In contrast, seven of the apoptosis-associated miRNAs, including miR-153, miR-155, miR-182, miR-202, miR-204, miR-296 and miR-337, were obviously down-regulated. Of note, let-7 family members, including let-7a, let-7b and let-7c, showed a significant relationship with anisomycin (Fig. 4C). qPCR revealed the trend similar to the microarray data, showing that the most up-regulated miRNA was *miR let-7c* (Fig. 4D).

**Figure 5:**
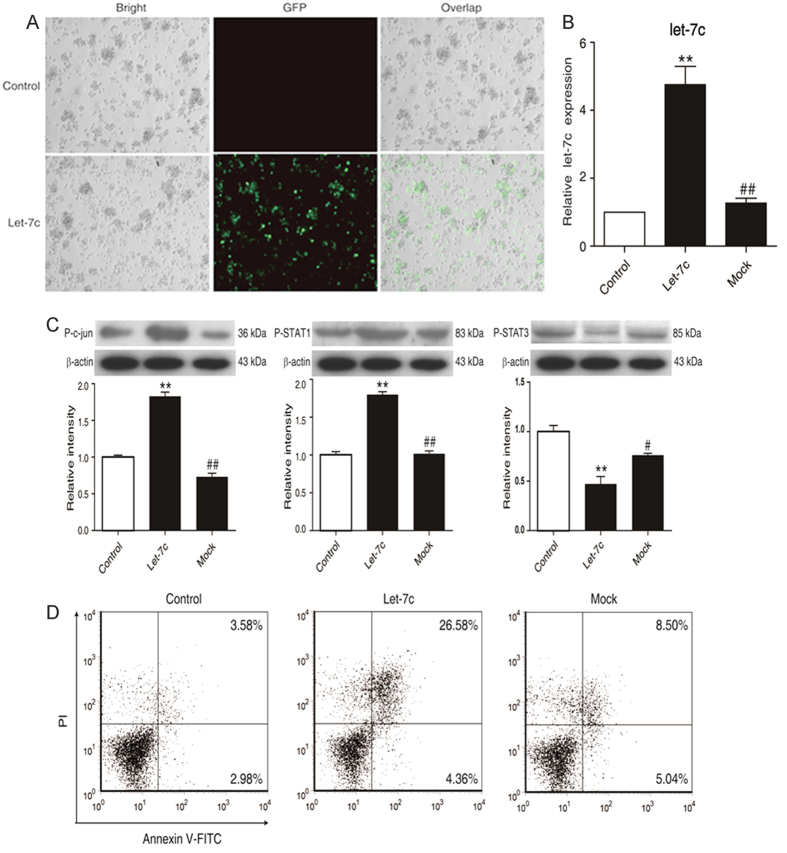
miRNA let-7c overexpression facilitates the anisomycin-stimulated apoptosis in Jurkat T cells. Jurkat T cells were electro-transfected with 2 μg of the let-7c overexpression vector with GFP marker (let-7c) or 2 μg of the miR-Negative Control vector (mock) for 24 h or 48 h. (**A**) GFP expression in Jurkat T cells. (**B**) The expression of *miR let-7c* in the transfected cells was measured by real-time qPCR. (**C**) The levels of phospho-c-jun, phospho-STAT1 and phospho-STAT3 were analyzed by Western blot. (**D**) The apoptotic rate of the treated cells was assayed by flow cytometry. The data are presented as the mean ± SD of three independent experiments. *p < 0.05, **p < 0.01 *vs*. the untreated control, ^#^*p* < 0.05, ^##^*p* < 0.01 *vs*. the let-7c overexpression group.

To further explore the role of the *miR let-7c* augment in the anisomycin-treated cells, an overexpressed vector containing *miR let-7c* was electro-transfected into Jurkat T cells with the transfection rate of 60–70% (Fig. 5A). Consequently, *miR let-7c* facilitated the significant enhancement of AP-1 and STAT1 in the anisomycin-induced apoptosis. On the contrary, STAT3 was suppressed with the enhancement of *miR let-7c* (Fig. 5B,C). The foregoing alterations led to the occurrence of the cell apoptosis (Fig. 5D). These results suggest that the miRNA let-7c regulates AP-1/STAT1/STAT3 to mediate the anisomycin-induced apoptosis.

### miRNA let-7c mediates the anisomycin-triggered apoptosis *via* linking JNK to the AP-1/STAT1/STAT3/Bim/Bcl-xL/Bak/Bax signaling

**Figure 6:**
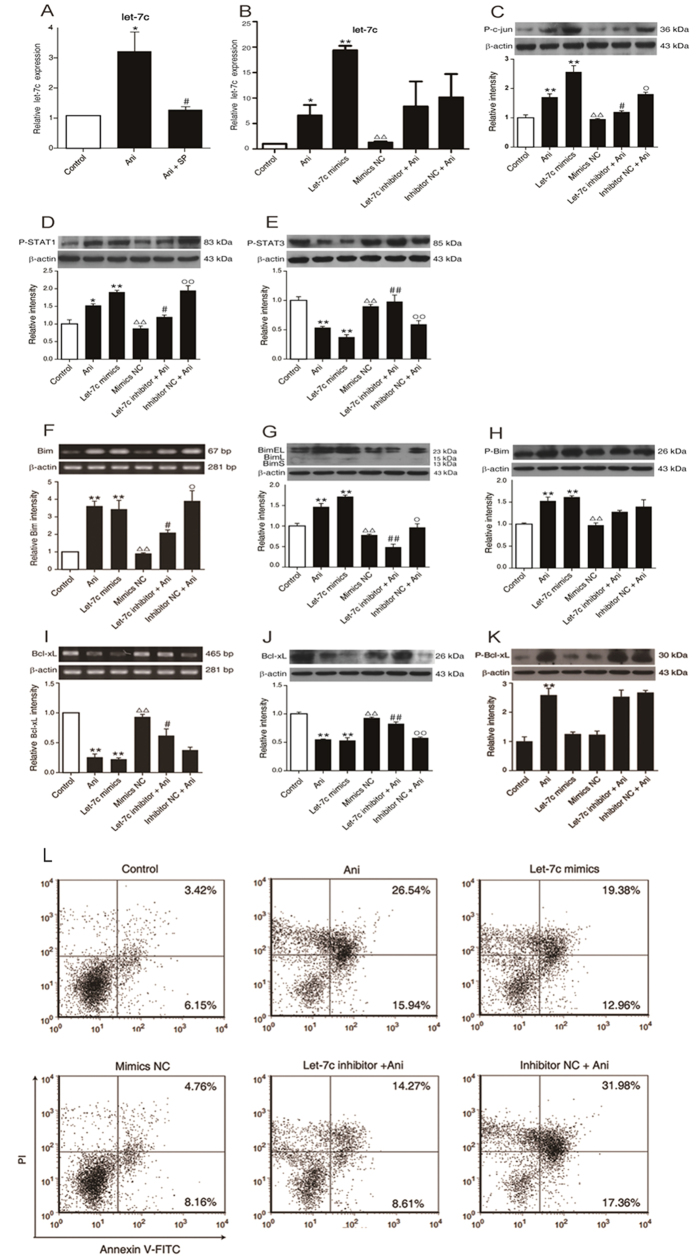
Blocking miRNA let-7c inhibits the anisomycin-induced apoptosis of Jurkat T cells. (**A**) Jurkat T cells were treated with or without 40 ng/ml of anisomycin for 24 h in the presence or absence of 10 μM SP600125. Afterwards, *miR let-7c* expression was measured by real-time qPCR. (**B**) After transfected with the let-7c mimics, let-7c mimics-negative, let-7c inhibitor or let-7c inhibitor-negative, the cells were exposed to or not to 40ng/ml anisomycin for 24 h. For the comparison, the untreated group and the 40 ng/ml anisomycin group were also set. Then, *miR let-7c* levels were measured by real-time qPCR. (**C**–**K**) The cells were treated as described above and the levels of P-c-jun, P-STAT1, P-STAT3, Bim, P-Bim, Bcl-xL and P-Bcl-xL were measured by Western blotting, and the β-actin expression was used for the normalization, in which the mRNA levels of Bim (**F**) and Bcl-xL (**I**) were simultaneously determined by RT-PCR and qPCR, respectively. (L) After transfected with the let-7c mimics, let-7c mimics-negative, let-7c inhibitor or let-7c inhibitor-negative, the cells were analyzed by flow cytometry for their apoptotic rate. The data are presented as the mean ± SD of three independent experiments. *p < 0.05 and **p < 0.01 *vs*. the untreated control, ^#^*p* < 0.05 and ^##^*p* < 0.01 *vs*. the 40 ng/ml of anisomycin group, ^Δ^*p* < 0.05 and ^ΔΔ^*p* < 0.01 *vs*. the let-7c mimics group, °*p* < 0.05 and °°*p* < 0.01 *vs*. the let-7c inhibitor group.

As shown in Fig. 6, the level of *miR let-7c* was remarkably increased in the cells treated with 40 ng/ml anisomycin, which could be rescued using SP600125, suggesting that downstream of JNK1/2 activated by anisomycin is *miR let-7c* (Fig. 6A). When 50 nM of *miR let-7c* mimics was transfected into the cells, the level of *miR let-7c* was increased up to about 20-fold as compared to the control, which might be abrogated by the let-7c mimics NC (negative control) instead of *miR let-7c*. Furthermore, the anisomycin-induced *miR let-7c* enhancement could be reversed by transfecting the cells with the *miR let-7c* inhibitor before the anisomycin treatment, but the *miR let-7c* inhibitor NC had no effect on this change (Fig. 6B). These further support the foregoing findings.

Simultaneously, the expressions of both phosphorylated c-jun (AP-1) and phosphorylated STAT1 were significantly up-regulated by anisomycin, which could be reversed by the transfection of a *miR let-7c* inhibitor, nor a *miR let-7c* inhibitor NC. Consistent with this change, the levels of the both were also increased after the transfection of the *miR let-7c* mimics, compared to either the control or the *miR let-7c* mimics NC group (Fig. 6C,D). On the contrary, the phosphorylation of STAT3 was inhibited by anisomycin. Similarly, the *miR let-7c* mimics could also suppress the phosphorylation of STAT3. The anisomycin-induced STAT3 phosphorylation could be blocked by transfecting the cells with the *miR let-7c* inhibitor before the anisomycin treatment (Fig. 6E).

The similarly treated cells were used to observe the changes of Bim and Bcl-xL. Consequently, the alteration of Bim mRNA (Fig. 6F) was consistent with that of its total protein (Fig. 6G). For both the unphosphorylated and phosphorylated Bim, the tendency is similar as to the alterations in the phosphorylated c-jun and the phosphorylated STAT1 (Fig. 6F–H). The alteration of Bcl-xL mRNA (Fig. 6I) was also consistent with that of its total protein (Fig. 6J). Similar to the change of the phosphorylated STAT3, the unphosphorylated Bcl-xL was decreased by anisomycin or the *miR let-7c* mimics, which could be rescued by the *miR let-7c* inhibitor (Fig. 6J). Interestingly, anisomycin could promote the Bcl-xL phosphorylation, which was not influenced by the *miR let-7c* inhibitor (Fig. 6K). This suggests that anisomycin might increase the phosphorylation of Bcl-xL *via* other molecules.

Following the activation of c-jun and STAT1, and the inhibition of STAT3 with the enhancement of Bim and the attenuation of Bcl-xL, the apoptotic rate of the cells was markedly increased by anisomycin. If the cells were transfected with the *miR let-7c* inhibitor before treated with anisomycin, this elevated apoptosis could be rescued, but the *miR let-7c* inhibitor NC had no obvious effect. Likewise, the *miR let-7c* mimics augmented the apoptotic number of the cells, but not the *miR let-7c* mimics NC (Fig. 6L).

**Figure 7:**
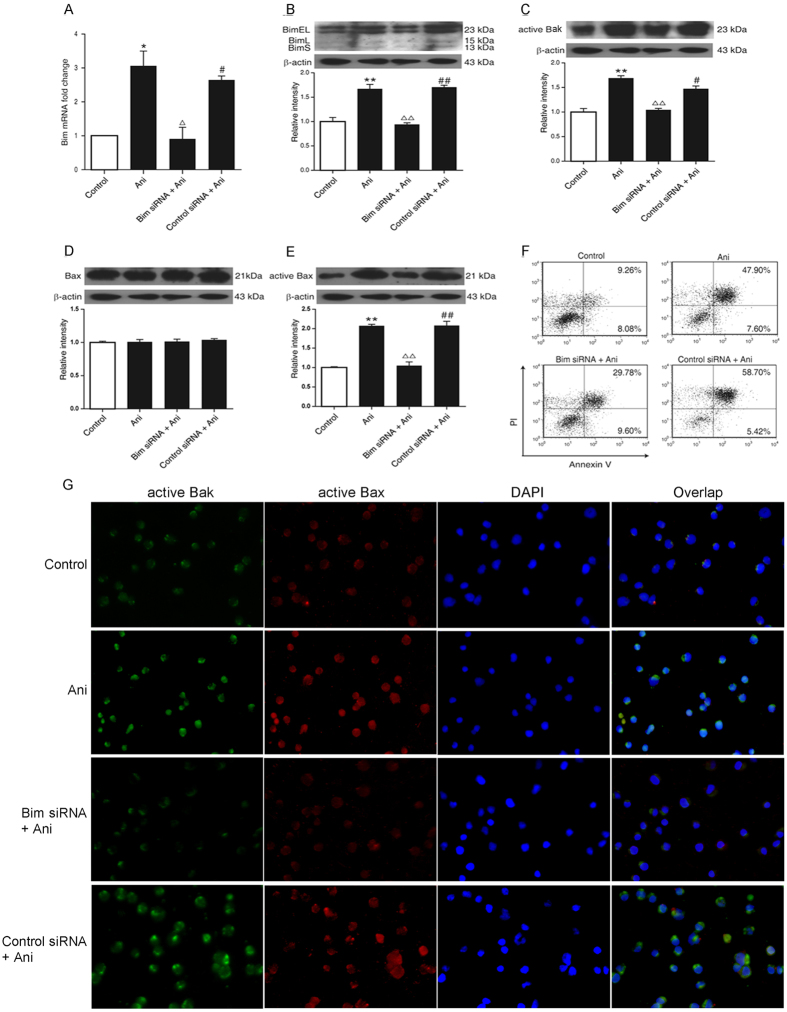
Role of Bim in the anisomycin-induced apoptosis in Jurkat T cells. The Jurkat T cells were transfected with 100 nM of Bim-targeting siRNA or control siRNA for 24 h to knockdown the *bim* gene. Then, 40 ng/ml of anisomycin was added into the cells for 24 or 48 h. (**A**) The Bim mRNA expression was evaluated by the real-time qRT-PCR. (**B**) The level of Bim protein was determined by Western blotting. (**C**–**E**) The levels of active Bak, Bax and active Bax were also measured through Western blotting. (**F**) The apoptotic proportion of the treated cells was analyzed by flow cytometry. (**G**) *In situ* immunofluorescence staining was performed for the changes of the active Bak and Bax in the treated cells (×200). The data are presented as the mean ± SD of three independent experiments. *p < 0.05 and **p < 0.01 *vs*. the untreated control, ^Δ^*p* < 0.05 and ^ΔΔ^*p* < 0.01 *vs*. the anisomycin group, ^#^*p* < 0.05 and ^##^*p* < 0.01 *vs*. the Bim siRNA group.

The anisomycin-promoted Bim mRNA expression was reversed by knocking down the *bim* gene in the cells (Fig. 7A). The alterations in Bim, active Bak and active Bax proteins showed the consistent tendency (Fig. 7B–E). The knockdown of the *bim* gene could impede anisomycin to boost the apoptosis of the cells (Fig. 7F). This could be further supported through the *in situ* immunofluorescence staining (Fig. 7G). These results demonstrate that Bim mediates the anisomycin-induced apoptosis by the active Bak and Bax.

## Discussion

Most of reports support that activation of the JNK and/or p38 can promote the cell apoptosis^31^,^32^,^33^,^34^,^35^,^36^,^37^,^38^. Our studies indicate that the activation of JNK1/2 is more predominant than that of p38 in the anisomycin-induced apoptosis. As regards to the mechanism of the apoptosis in the anisomycin-treated Jurkat T cells, the JNK activates the apoptotic signaling by directly modulating the activities of the mitochondrial pro- and anti-apoptotic proteins through the phosphorylation events^31^. Lei *et al*. found that the JNK phosphorylates two members of the BH3-only subgroup of Bcl2-related proteins (Bim and Bmf) during the UV-induced Bax-dependent apoptosis^32^. Recently, Abayasiriwardana *et al*. showed that anisomycin induced the rapid JNK-dependent phosphorylation of Bim in mesothelioma cells^33^. In our study, not only the proapoptotic Bim, but also the anti-apoptotic Bcl-xL was phosphorylated by JNK1/2 during the anisomycin-induced cell apoptosis. The JNK-mediated phosphorylation of Bcl-xL decreased its antiapoptotic activity. That is, Bcl-xL exerts the anti-apoptotic action, but rather the phosphorylated-Bcl-xL lacks its anti-apoptotic one. This is supported by the reported findings that Bcl-xL heterodimerizes with the proapoptotic molecules, such as Bim, Bak and Bax, to suppress their activity, while the serine 62 phosphorylation of Bcl-xL abolishes its anti-apoptotic effect. For example, B cell receptor crosslinking induces the serine 62 phosphorylation by JNK with the subsequent degradation of Bcl-xL *via* the ubiquitin proteasome pathway^34^. Additionally, our data demonstrated that the anisomycin-induced cell apoptosis was rescued following the reduction of Bim by knocking down *bim* gene or the inhibition of Bim activity with the JNK inhibitor. These findings strongly prove that anisomycin promotes the apoptosis in Jurkat T cells through the JNK-dependent activation of Bim/Bcl-xL.

In other aspect, JNK signaling also leads to an increase of pro-apoptotic gene expressions by transactivating the specific transcription factors, such as c-Jun^31^. It has been observed that the phosphorylated JNK translocates toward the nucleus where it phosphorylates c-Jun, causing the formation of AP-1^35^,^36^. A recent finding shows that the c-Jun/AP-1 is rapidly activated upon stimulation with anisomycin in human hepatoma cells^37^. Our results further display that both transcriptional and DNA-binding activities of AP-1 are enhanced in parallel with the phosphorylation of STAT1 by anisomycin in the JNK-dependent manner. It is now largely admitted that Bim is the transcriptional target of AP-1 and STAT1^38^. Interestingly, there is sufficient evidence in neuronal apoptosis that the Bim mRNA is induced by a mechanism dependent on the JNK/AP-1 pathway^39^. These analogous results in neural cell death enkindle us to associate Bim with the JNK/AP-1 and JNK/STAT1 pathways. Although potential connection between Bim and AP-1 or STAT1 needs to be proved, we did observe the prominently enhanced Bim mRNA/protein/activity/function in the anisomycin-induced cell apoptosis, following the elevation of AP-1 and STAT1 activities by activating the JNK signaling pathway. Accordingly, anisomycin contributes to the apoptosis in Jurkat T cells through the JNK/AP-1-STAT1/Bim signaling axis.

In the context of roles of the JNK in apoptotic signaling pathways, how does it cause the reduction of Bcl-xL in the process of the anisomycin-induced apoptosis? A reasonable speculation is that Bcl-xL might be negatively regulated by some proapoptotic miRNAs. To confirm it, we screened 48 miRNAs involved in the cell apoptosis for the first time. Consequently, *miR let-7c* is significantly up-regulated after the anisomycin treatment. It has been demonstrated that the let-7 family members of miRNAs negatively regulate the Bcl-xL expression in the human hepatocellular carcinomas to induce apoptosis in cooperation with sorafenib, an anti-cancer drug^40^. Additionally, Qin *et al*. showed recently that the let-7c expression was markedly up-regulated in the ox-LDL-induced apoptosis of endothelial cells (ECs) and that the Bcl-xL was the direct target of let-7c in ECs^41^. However, we notice that the negative regulation of let-7 miRNA on Bcl-xL expression is only at the post-transcriptional level, but not at the transcriptional level. Our data present for the first time that let-7c can down-modulate the phosphorylated STAT3, but that the block of let-7c can prevent anisomycin from down-modulating the phosphorylated STAT3. Based on the report that STAT3 up-regulates the Bcl-xL expression at the transcriptional level. This study proposes that let-7c down-regulates the expression of Bcl-xL *via* inhibiting STAT3 activation in the anisomycin-induced apoptosis. Moreover, our results indeed show that the negative regulation of let-7c on STAT3 activity is in a JNK-dependent manner. However, the mechanism by which JNK regulates let-7c in the anisomycin-elicited apoptosis remains to be clarified.

**Figure 8:**
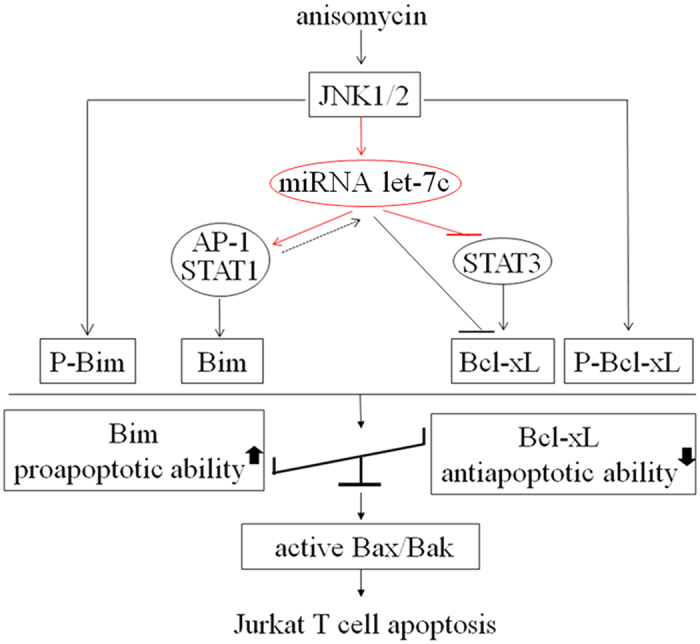
Proposed potential signaling pathway for the anisomycin-induced apoptosis in Jurkat T cells. The black lines or arrows display the literature-revealed signaling pathway, which is also proven strongly by our using anisomycin in the Jurkat T cell model, whereas the red lines or arrows show our result-revealed novel signaling connection.

Additionally, how does JNK impact the enhancement of Bim in the process of the anisomycin-induced apoptosis? A transcription factor AP-1 is a strong positive regulator of many miRNAs, including miR-21, miR-199a-5p and miR-203^42^,^43^,^44^. We found that let-7c positively regulated both AP-1 and STAT1, but negatively did Bcl-xL in Jurkat T cells. Talotta *et al*. showed that miR-21 positively regulated the AP-1 activity by targeting its negative regulator PDCD4, suggesting that miR-21 is both the target and the regulator of AP-1 in the RAS-mediated transformation^45^. Our results reveal for the first time that let-7c may up-regulate the activities of both the AP-1 and the STAT1 to accelerate the phosphorylation of Bim, functioning as a bridge to pass JNK1/2 signaling onto the AP-1/STAT1/STAT3, thereby activating the AP-1/STAT1, and inhibiting the STAT3. It has been reported that AP-1/STAT1 can augment the Bim level, whereas let-7c directly diminishes the level of STAT3. Moreover, the current study indicates that let-7c is able to attenuate the Bcl-xL level through decreasing STAT3 activity. Furthermore, Bim can activate Bax and Bak, thereby initiating the cell apoptosis (Fig. 8). Therefore, we can conclude that the miRNA let-7c links the JNK1/2 to the AP-1/STAT1/STAT3 signaling, and activates the Bak/Bax through breaking the balance between the anti-apoptotic Bcl-2 family protein and the proapoptotic BH3-Only protein, finally resulting in the anisomycin-triggered apoptosis of Jurkat T cells. This provides a novel insight into the mechanisms by which anisomycin causes the apoptosis of the cancer cells. Considering its low dose and high efficacy as well as the low adverse effect profile, anisomycin is relatively promising to be developed and potentially applied to the clinical treatment of the human acute lymphocyte leukemia.

## Methods

### Cell and culture

Jurkat T cells (China Center for Type Culture Collection Wu Han University) were cultured at 37 °C in 5% CO2 in RPMI 1640 complete culture medium containing 10% (v/v) fetal bovine serum (Gibco BRL, Grand Island, NY, USA).

### Nuclear transfection with the a *miR let-7c* overexpression vector

1 × 10^6^ of Jurkat T cells per sample were resuspended in 100 μl room-temperature Nucleofector® Solution (VCA-1003) (Lonza Amaxa, Germany) according to the manufacturer’s instructions. The cell suspension was combined with 2 μg of the let-7c overexpression vector or 2 μg of the miR-NC vector (B044) (GenePharma, ShangHai, China). Then, the cell/DNA suspension was transfected into Jurkat T cells with Nucleofector® Program X-01. The cells were analyzed at 6–24 h post nuclear transfection for transfection rate, qPCR and Western blot analysis. Forty eight hours after nuclear transfection, the cells were harvested for flow cytometry.

### siRNA transfection

Jurkat T cells were transfected using 100 nM of Bim-targeting siRNA or 100 nM of negative control siRNA (siN05815122147) enwrapped with lipofectamine 2000 (Invitrogen, Carlsbad, CA, USA) for 24 h in accordance with the manufacturer’s instructions, and then treated with 40 ng/ml of anisomycin. Twenty-four hours after the treatment, the cells were harvested for Real time-quantitative PCR (qPCR) and Western blotting, and at 48 h after the treatment for Flow Cytometry. The Bim siRNA sequence is 5′-TGGGTAGGCCTTTGTACTTAAdTdT-3′ (Aguilo *et al*., 2014).

### miRNA transfection

Jurkat T cells were transfected using 50 nM of let-7c mimics or 100 nM of the let-7c inhibitor or their corresponding negative control miRNA (RiboBio, Guangzhou, China) mixed with lipofectamine 2000 (Invitrogen, Carlsbad, CA, USA) according to the manufacturer’s instructions. The cells were transfected for 6 h before addition of 40 ng/ml anisomycin for qPCR, Western blotting and Flow Cytometry, respectively.

### Semi-quantitative RT-PCR

Jurkat T cells were treated for 2 to 4 h with the incremental concentrations of anisomycin (0, 5, 10, 20, 40, and 80 ng/ml) or with 40 ng/ml of anisomycin after the treatment with 10 μM SP60012 or 20 μM PD98059. Total RNA in the treated cells was extracted using Trizol reagent (Invitrogen, Carlsbad, CA, USA) following the manufacturer’s protocols. The primers were employed for the semi-quantitative PCR analysis. Bim forward, 5′-GCAGATATGCGCCCAGAGAT-3′ and reverse, 5′-AAGCGTTAAACTCGTCTCCGATA-3′; Bcl-xL forward, 5′-GTGCGTGGAAAGCGTAGACA-3′ and reverse, 5′-CAGCCAAGGTGACCCATTAC-3′; β-actin forward, 5′-AACAGTCCGCCTAGAAGCAC-3′ and reverse, 5′-CGTTGACATCCGTAAAGACC-3′.

### Real time-quantitative PCR

qPCR was performed on a Bio-Rad CFX96 PCR system (Bio-Rad, USA). The used primers are shown in Table 1. Real-time qPCR was performed with the following procedure: 95 °C for 15 min, and 40 repeats at 95 °C for 10 s, 60 °C for 20 s and 72 °C for 10 s. The 2^−△△Ct^ method was performed for the relative quantification of the mRNA expression.

### Western blot

Jurkat T cells were treated with 40 ng/ml of anisomycin at the different time (0, 0.5, 1, 2, 3, 4, and 6 h) or with the incremental doses of anisomycin (0, 5, 10, 20, 40, and 80 ng/ml) for 1 to 2 h, concomitantly in the presence or absence of 10 μM SP60012 or 20 μM PD98059 or 20 μM SB203580. The blotted PVDF membranes were incubated overnight with anti-ERK1/2, anti-P-ERK1/2, anti-p38, anti-P-p38, anti-JNK1/2, anti-P-JNK1/2, anti-Bim, anti-P-Bim, anti-β-actin (Cell Signaling, Danvers, MA, USA), anti-P-Bcl-xL (Santa Cruz Biotechnology, Santa Cruz, CA, USA), anti-STAT1, anti-STAT3, anti-c-jun, anti-P-STAT1, anti-P-STAT3, anti-P-c-jun (Bioss Inc, Woburn, Massachusetts, USA), anti-Bak, anti-Bax (Abclonal, Cambridge, MA, USA) and active Bax (6A7) (Beyotime, Shanghai, China), respectively, and done with HRP-conjugated anti-goat IgG (Santa Cruz Biotechnology, Santa Cruz, CA, USA), HRP-conjugated anti-mouse IgG and HRP-conjugated anti-rabbit IgG (Cell Signaling, Danvers, MA, USA), respectively.

### DNA ladder

Jurkat T cells were treated with 40 ng/ml of anisomycin at the different time (0, 0.5, 1, 2, 3, 4, and 6 h) or with various concentrations of anisomycin (0, 5, 10, 20, 40, and 80 ng/ml) for 6 h, concomitantly in the presence or absence of 10 μM SP60012. After the treatments, their fragmented DNAs were extracted and purified using Apoptotic DNA Ladder Kit (Beyotime, Shanghai, China) according to the manufacturer’s instructions.

### Annexin-V/PI staining

Jurkat T cells were treated with 40 ng/ml of anisomycin at different time (0, 0.5, 1, 2, 3, 4, and 6 h) or with the increasing doses of anisomycin (0, 5, 10, 20, 40, and 80 ng/ml) for 6 h or 24 h, concomitantly in the presence or absence of 10 μM of SP60012 or 20 μM of PD98059. The treated cells were incubated in binding buffer containing 5 μl of Annexin V-FITC and 10 μl of propidium iodide (PI) (KeyGEN Biotech, Nanjing, China) for 15 min at room temperature for flow cytometry analysis.

### Apoptosis-associated miRNA array

The miRNA levels in Jurkat T cells treated with 40 ng/ml of anisomycin were determined by the Apoptosis-Associated miRNA Plate Array (Signosis, Sunnyvale, CA, USA) following the manufacturer’s instructions. 10–30 μg of the extracted total RNA was utilized for hybridization in the 96-well plate which was pre-coated with the oligo mix, including a pair of unique oligos that hybridize side-by-side to the specific target miRNA, the universal capture oligo and the biotin-labeled oligo. Streptavidin-HRP conjugate was used for the detection of the miRNA expression. The chemiluminescence of each well was determined with the 1420 Victor Multilable Counter (PerkinElmer, USA).

### TF reporter array

The analysis of the transactivation of seven transcription factors (TFs) was simultaneously performed using the TF Reporter Plate Array I (Signosis, Sunnyvale, CA, USA) according to the manufacturer’s instructions. The transfected cells were treated with anisomycin at the different doses of 10, 20, 40, and 80 ng/ml or with 40 ng/ml of anisomycin after the pretreatment with 10 μM SP60012. Total RNA in the treated cells was extracted using Trizol reagent. Subsequently, cDNA synthesis and Plate hybridization were performed. Finally, the chemiluminescence of each well was determined with a 1420 Victor Multilable Counter.

### Electrophoresis mobility shift assay

EMSA was completed using the AP-1 EMSA Kit (Signosis, Sunnyvale, CA, USA), based on the protocol provided by the manufacturer. Briefly, binding reactions containing 2–5 μg of nuclear protein, 2 μl of 10 × binding buffer, 1 μg poly (dI:dC), 1–4 μl non-Rnase water and 1 μl of oligonucleotide probe were kept at room temperature for 30 min. Protein-DNA complexes were separated on a 6% non-denaturing acrylamide gel, transferred to positively charged nylon membranes, and then cross-linked in a Stratagene UV cross-linker. Streptavidin-HRP was added to the NC membrane, and the blots were developed by ECL.

### Immunofluorescence staining

Jurkat T cells were transfected using Bim-targeting siRNA, and treated with anisomycin as described in the “miRNA transfection” section. The treated cells were incubated with rabbit anti-human Bak, rabbit anti-human Bax (1:100) (Abclonal, Cambridge, MA, USA) and mouse anti-human 6A7 (1:500) (Beyotime, Shanghai, China) antibodies at 4 °C overnight. Subsequently, the cells were incubated with an Alexa Fluor 488 conjugated goat anti-rabbit IgG antibody (1:1,000 diluted in the blocking solution) (Cell Signaling, Danvers, MA, USA) at room temperature for 1 h.

### Statistical analysis

Data are presented as means ± S.D. Statistical differences were examined using one-way ANOVA followed by the multiple comparison tests or the unpaired two-tailed Student’s *t* test. *p*-values < 0.05 are considered to be the significant difference.

## Additional Information

**How to cite this article**: Zhou, Z. *et al*. microRNA let-7c is essential for the anisomycin-elicited apoptosis in Jurkat T cells by linking JNK1/2 to AP-1/STAT1/STAT3 signaling. *Sci. Rep*. **6**, 24434; doi: 10.1038/srep24434 (2016).

## Figures and Tables

**Table 1:** Sequences of qPCR primers.

miRNA	Forward primer	Reverse primer
hsa-mir-let-7chsa-mir-26	Forward:5′-GGG TGAGGTAGTAGGTTGT-3′Forward:5′-GGG TTCAAGTAATCCAGGA-3′	Reverse:5′-CAGTGCGTGTCGTGGAGT-3′Reverse:5′-CAGTGCGTGTCGTGGAGT-3′
hsa-mir-133b	Forward:5′-GGG TTTGGTCCCCTTCAAC-3′	Reverse:5′-CAGTGCGTGTCGTGGAGT-3′
hsa-mir-144	Forward:5′-GGG GGATATCATCATATAC-3′	Reverse:5′-CAGTGCGTGTCGTGGAGT-3′
hsa-mir-193aa	Forward:5′-GGG TGGGTCTTTGCGGGCG-3′	Reverse:5′-CAGTGCGTGTCGTGGAGT-3′
hsa-mir-296	Forward:5-GGG AGGGCCCCCCCTCAA-3′	Reverse:5′-CAGTGCGTGTCGTGGAGT-3′
